# The Impact of Personality and Lifestyle Change on Distress During the COVID-19 Pandemic

**DOI:** 10.1525/collabra.19525

**Published:** 2021-03-03

**Authors:** Caroline E. Balling, Skye C. Napolitano, Sean P. Lane, Douglas B. Samuel

**Affiliations:** 1 Department of Psychological Sciences, Purdue University (IN)

**Keywords:** five-factor model, covid-19, coronavirus, personality, distress, coping

## Abstract

The COVID-19 pandemic provided a unique opportunity for quantifying the impact of Five Factor Model personality domains (i.e. neuroticism, extraversion, openness, agreeableness, and conscientiousness) and COVID-related lifestyle changes on psychological distress. To examine these relationships, we designed and preregistered the present study (https://osf.io/qfw9h). We assessed a large, heterogeneous sample including undergraduates, graduate students, faculty, and staff of a large, public, Midwestern university (*n* = 1055) to ascertain whether personality domains uniquely predicted distress in response to COVID-19 shelter-in-place orders. This was a three-panel study in which the same potential participant pools were invited to participate at each survey announcement. Data collection occurred between early March through late May 2020, from within days of local shelter-in-place order onset to within days of reaching 100,000 COVID-related deaths in the USA. Domain and distress scores were determined from self-reported ratings on the Big Five Inventory and the 21-Item Depression Anxiety and Stress Scales, respectively. Participants also reported personal experiences with six COVID-specific lifestyle impacts: insufficient outdoor or indoor living space, job insecurity, income insecurity, or taking care of or homeschooling school-aged children during working hours. Zero-order correlations revealed that all personality domains except openness had statistically significant correlations with distress, and all correlations were negative except for that of neuroticism. When entered simultaneously, neuroticism was the predominant risk factor of distress that held across all preregistered and exploratory analyses. Our expectation that extraversion would be negatively associated with distress was not supported broadly, while agreeableness was a unique potential risk factor (though this effect was mostly limited to exploratory analyses). The results especially highlight the link between employment and income uncertainty with psychological distress, while also identifying insufficient indoor and outdoor space as potential risk factors. We hope these findings inform future public health action and further emphasize the utility of personality trait models in general.

The Coronavirus Disease 2019 (COVID-19) triggered a global pandemic with over 82.2 million reported cases and 1.8 million reported deaths worldwide by the beginning of 2021 (World Health Organization, 2020b). As the pandemic escalated, shelter-in-place and stay-at-home orders were initiated across the United States beginning in March 2020. Daily quality of life declined for most people during the pandemic, particularly within the confines of these stay-at-home orders (Williams et al., 2020). These difficulties were potentially worsened by pandemic-related stressors such as social isolation, loss of income, not having a safe or comfortable home environment, or homeschooling children, as well as individuals or their loved ones having health conditions that could exacerbate COVID-19 risk (e.g., immunosuppression, asthma, chronic upper respiratory condition). Additionally, those with access to television, internet, or other news sources were flooded by constant media coverage of the pandemic, and this likely exacerbated feelings of fear and anxiety.

The combination of COVID-related stressors and risk factors has contributed to elevated feelings of stress, fear, and other negative emotions compared to life-as-usual. In August of 2020, the CDC released a report showing that nearly 24% of young adults had contemplated suicide during June 2020 of the pandemic, highlighting the mental health crisis and illustrating the need to better determine who is at the greatest risk. Other research has found that social distancing, social isolation, and loss of routine, working, or a sense of safety are associated with symptoms of stress, anxiousness, grief, and even post-traumatic stress during the pandemic (e.g. Bo et al., 2020; Galea et al., 2020; Pfefferbaum & North, 2020; Wallace et al., 2020). There has been widespread awareness of these potential mental health consequences, with the WHO and Center for Disease Control (CDC) dedicating web pages to mental health during the COVID-19 pandemic specifically (Centers for Disease Control and Prevention, 2020; World Health Organization, 2020a).

The pandemic represented a uniquely pervasive and severe stressor at the global level, and research into the human reaction to such a disaster is useful for broader understanding of the psychological constructs underlying stress and resilience. Because all individuals were exposed to a substantial stressor at the same time, the pandemic provided a unique natural experiment for quantifying the impact of factors such as personality traits and lifestyle changes on distress. In the present study, we chose to assess personality traits because research has made clear that they are risk factors for increased stress response, protective factors against increased distress, and intimately related to coping style (Clark et al., 1994; Kotov et al., 2010; McCrae & Costa, 2013). Although some research into COVID-19 and personality traits has been conducted, these studies have not focused on the link between traits and distress during the pandemic, instead examining topics such as compliance with pandemic regulations or the possibility of personality change during the pandemic (e.g. Carvalho et al., 2020; Götz et al., 2020; Hardin et al., 2020; Sutin et al., 2020).

There is broad agreement that general personality traits can be organized into five broad dimensions (John et al., 2008). These dimensions have been called the Big Five or the Five Factor Model of personality (FFM; McCrae & Costa, 2013). This model boasts generalizability across cultures, languages, the lifespan, and informant source, as well as tracible genetic correlates (Kotov et al., 2010; Power & Pluess, 2015; Roberts & DelVecchio, 2000; Schmitt et al., 2007). It includes five broad, bipolar dimensions: neuroticism vs. emotional stability, extraversion vs. detachment, openness vs. closedness to one’s experience, agreeableness vs. antagonism, and conscientiousness vs. undependability.

Neuroticism represents an individual’s global tendency to experience negative affect such as fear, sadness, or anger. Those higher in neuroticism often display impulsivity, low self-esteem, and poor coping skills. In contrast, extraversion in associated with the experience of positive emotion, and those higher in extraversion are often warm, assertive, and excitement-seeking. Conscientiousness is associated with organization, planning, achievement, and self-control. Openness relates to creativity, intellect, curiosity, and a connection to inner feelings. Those higher in agreeableness are often characterized as altruistic, empathic, and trusting.

Research has repeatedly indicated that personality traits are reliable risk factors for the experience of depression, anxiety, and stress (Pocnet et al., 2017; Vollrath, 2001). Specifically, elevated neuroticism is implicated in internalizing problems, while higher extraversion and conscientiousness are thought of as a buffer against them (Kotov et al., 2010; Naragon-Gainey & Simms, 2017). One meta-analysis concluded that experiencing clinically elevated psychiatric symptomatology was associated most with high neuroticism, followed by low conscientiousness, low extraversion, and low agreeableness, while there was no broad association with openness (Malouff et al., 2005). Similarly, a mega-analysis of 66 meta-analyses concluded that all depressive, anxiety, and substance use disorders were most reliably associated with increased levels of neuroticism and decreased levels of conscientiousness (Kotov et al., 2010). Altogether, neuroticism, conscientiousness, and extraversion are the traits most consistently associated with clinically significant psychological difficulties.

We approached the current investigation with the understanding that personality and episodic psychiatric distress are closely related. With this well-evidenced association in mind, we examined the role of personality as a potential accelerant for the transient symptoms of distress experienced within a heterogeneous community and in response to a severe, universally-shared stressor: the COVID-19 global pandemic.

Past research into large-scale traumas offers some insight into the impacts of such a stressor. Following 9/11, 44% percent of adults in the United States reported one or more clinically relevant symptoms of distress, while 90% endorsed at least one symptom to some degree (Schuster et al., 2001). Several studies concluded that psychological distress was not limited to those who experienced the trauma of 9/11 directly, such as by living in New York City or losing a loved one in the attacks (Marshall et al., 2007; Silver et al., 2002, 2005). This unanticipated phenomena has been observed in other countries following other mass-casualty events as well (Bleich et al., 2003; Marshall et al., 2007; Rubin et al., 2005). Such findings challenge the traditional conceptualization of trauma in which the severity of psychological consequences is positively related to proximity to the disaster. Thus, we would expect many people to experience a negatively-valanced psychological response to COVID-19 whether or not they closely experienced the illness, death, or poverty left in the wake of the pandemic. Several studies following 9/11 concluded that it was partially the consumption of media coverage that predicted both short- and long-term stress response (Ahern et al., 2002; Marshall et al., 2007; Schlenger et al., 2002). Indeed, there has been no shortage of media coverage during the COVID-19 pandemic, suggesting that the degree to which individuals engage in COVID-related media consumption may impact their levels of distress.

Alternatively, Silver and colleagues concluded that the detrimental psychological effects of 9/11 were *not* associated with amount of exposure or loss related to the trauma—but instead with type of coping strategies utilized (Silver et al., 2002). Clark et al. (1994) posited over two decades ago that personality influences the ways in which individuals perceive, react, and cope with stressful life events, particularly when inspecting the traits of neuroticism and extraversion, and research has repeatedly corroborated this (Schwarzer, 2001; Straud et al., 2015). Several studies highlight that high extraversion, high conscientiousness, and low neuroticism are positively associated with positive coping mechanisms such as higher positive threat reappraisal (e.g. perceiving stressors as challenges rather than threats; considering adversity to be an opportunity for growth), decreased disengagement responses (e.g. lower rates of withdrawal, denial, or substance use), and the positive appraisal of potential resources such as social support (Carver & Connor-Smith, 2009; Penley & Tomaka, 2002; Vollrath, 2001).

Although considerable research has been conducted regarding psychological distress following a massive, wide-reaching stressor, very little of it has addressed personality’s role in these processes. One study found that neuroticism was positively associated with short- and long-term post-9/11 distress, while levels of extraversion, conscientiousness, agreeableness, and openness were negatively related to distress (Brow & Silver, 2009). The latter four domains were positively associated with a range of adaptive responses including active coping and planning, as well as negatively associated with maladaptive reactions such as self-blame, denial, and behavioral disengagement (Brow & Silver, 2009).

Diathesis-stress models have long suggested that the effect of stressors on the development of psychiatric difficulties can be moderated by personality (Ingram & Price, 2010; Kopala-Sibley et al., 2016; Monroe & Simons, 1991). Kopala-Sibley et al. (2016) surveyed personality traits among mothers as a diathesis for depression following Hurricane Sandy—the second costliest hurricane in U.S. history. The study concluded that even after adjusting for lifetime history of depressive disorders, higher levels of stress following the disaster did predict higher levels of depressive symptoms, but this effect was only true for those high in neuroticism or low in extraversion. Despite repeated evidence that neuroticism and extraversion confer vulnerability or protection from psychopathology, the authors point out that there is a paucity of data examining whether personality traits actively increase sensitivity to life stress (Kopala-Sibley et al., 2016). Even among the studies that do study this, many are limited only to neuroticism as a predictor and depression as an outcome (e.g. Brown & Rosellini, 2011; Hutchinson & Williams, 2007; Kendler et al., 2004; Ormel et al., 2001).

We designed the current study with the expectation that most respondents would be experiencing substantial distress, and that lifestyle changes caused by the pandemic and stay-at-home orders would influence levels of this distress. Specific to our assessment, we predicted that insufficient outdoor or indoor living space, job insecurity, income insecurity, or taking care of or homeschooling school-aged children during working hours would exacerbate negative affect. It stands to reason that the presence of children while working from home could substantially impede personal and professional goals and expectations, while inadequate space inside and outside of the home could increase the discomfort and fatigue from staying at home. Further, for those under the constant threat or active implementation of COVID-related furloughs, budget cuts, and business closures, changes to income and job status would certainly increase feelings of distress from struggling to make ends meet.

Indeed, COVID-19 has caused significant disruption to daily life for all and has the substantial potential for financial strain, serious physical illness of self or others, and negative impact on mental health. Exploration into the personality characteristics and life circumstances that predict emotional responses to the pandemic is broadly relevant to the current pandemic and future public health crises that may occur. There is a particular need for research surveying the impact of all FFM traits as a risk factor for and predictor of general distress following an acute stressor. Despite a recent empirical assertion that levels of neuroticism slightly changed during the pandemic (Sutin et al., 2020), trait stability is a central and well-evidenced tenant of personality theory (e.g. Costa et al., 2019; McCrae & Costa, 1994; Roberts & DelVecchio, 2000). We would not expect personality traits to substantially change on a short-term basis. Thus, traits serve as a reasonable predictor for distress, and collected measures of personality are sufficient reflections of respondent baseline.

The present study offers a unique glimpse into the relationship between personality and distress when options for coping are limited and efforts to be forward-thinking could be overwhelmed by the comparative uncertainty of the future. Importantly, the confinement and long-term nature of the COVID-19 pandemic allows us to consider these questions in the context of potentially more extreme and unique impacts than have been previously studied. The circumstances of this study also permitted more ecological validity than researchers of psychology and personality typically have, such that it allowed a narrowing of extraneous variables. Specifically, for this unique period of time, participants were living under similar restrictions, such as staying at home, isolating from friends, uprooting routine, and closely monitoring their physical health. This study serves as an opportunity for a purer perspective of the role of personality given a more tightly controlled situation.

## Current Study

We assessed FFM personality domains, COVID-related lifestyle characteristics, and recent symptoms of distress among a heterogeneous sample of adults affiliated with a large public university in the Midwest. The pre-registered study included three phases of data collection between the beginning of March and the end of May, 2020. Individuals were permitted to participate in multiple phases of data collection.

We established several a priori hypotheses. The first was that varying levels of FFM personality traits would differentially predict psychological distress, with the presence of specific COVID-19 lifestyle changes independently modifying these relationships. In accordance with the aforementioned research, we predicted that neuroticism would account for the most unique variance in psychological distress, followed by extraversion, then conscientiousness. We predicted that neuroticism would have a positive relationship with distress, while the latter two would be negatively related to distress. We did not anticipate agreeableness and openness to uniquely account for variance. We also hypothesized that increased levels of COVID-related lifestyle stressors (e.g. school-aged children at home, income insecurity) would be associated with higher levels of psychological distress. Finally, we anticipated that these lifestyle factors would moderate (i.e. change) the statistically significant relationships between personality traits and distress, such that the presence of additional lifestyle stressors would amplify the potentially pernicious effects of personality (i.e., elevated neuroticism) and dampen the stress-buffering effects of higher extraversion and conscientiousness.

## Methods

The study was preregistered on OSF prior to the completion of data collection and human observation of the data. The preregistration can be accessed at the following link: https://osf.io/qfw9h (Balling et al., 2020). No directly identifiable information was collected from participants, so the study was exempt from review and approved by the affiliated university’s Institutional Review Board (IRB).

### Participants

Participants were recruited for the study using a two-pronged approach. First, the survey was posted to a large, Midwestern University website for students to complete for research credit in their Introduction to Psychology courses. Additionally, emails were sent on behalf of the research team to a variety of publicly-available email lists of students and employees from several colleges within the university. The emails explained the broad purposes of the research study, requested participation from any university-affiliated undergraduates, graduate students, faculty, and staff, and included a Qualtrics link to the study. Upon accessing the survey, participants read the informed consent and indicated whether or not they consented to participate in the study. If they consented, they proceeded to the Qualtrics survey. The median time to complete the survey was 11 minutes, 58 seconds.

**Figure 1.**
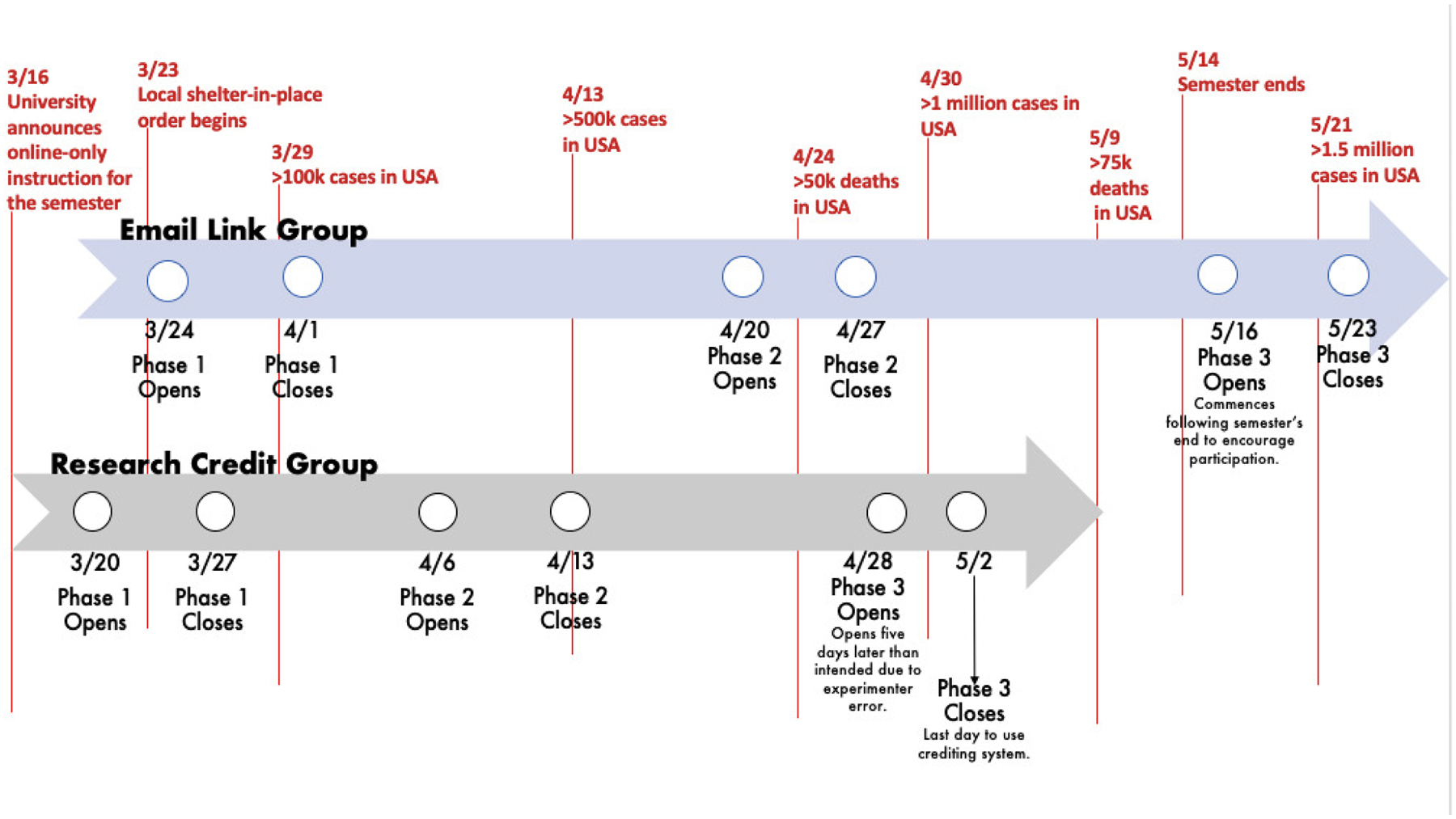
Coronavirus Disease 2019 and Data Collection Timelines.

Phase 1 of data collection occurred from March 20 through March 27, 2020 via the research crediting system (*n* = 192), and from March 24 through April 1, 2020 via the email link (*n* = 216). Phase 2 of data collection occurred from April 6 – 13 for the research crediting system (*n* = 86) and from April 20 – 27 for the email link (*n* = 114). Phase 3 was collected from April 23 – 30 for the research crediting system (*n* = 215) and May 16 – 23 for the email link (*n* = 232). An annotated timeline of data collection in the context of significant COVID-19 events can be found in Figure 1.

Following completion of the survey, participants recruited via email had the opportunity to enter a raffle for one of three $40 Amazon gift cards available at each phase. Participants could follow a link to a separate Qualtrics survey to enter their email address, and the identifying information from the raffle entry was not linked in any way to the participants’ main survey data. Following data collection, the investigators randomly selected three winners and emailed each of them an electronic Amazon gift card. Participants recruited through the university website were automatically granted class credit upon completion of the survey.

A total of 1,159 individuals consented to participate in the survey, but the sample for all presented analyses excluded individuals who demonstrated non-purposeful responding or minimally complete responses (*n* = 104). Specifically, two attention check items were embedded within the survey that indicated a specific response to rate that item (e.g., “select ‘moderately’ for this item), and participants were excluded from the analysis if they failed both of the attention checks (*n* = 23), took less than 5 minutes to complete the survey (*n* = 67; 5 minutes indicated less than 2.5 seconds spent on each item), and/or clicked through less than 97% of the Qualtrics survey (*n* = 58; 97% completion indicated only that a participant clicked through to the last page of the survey because no answers were required). Remaining responses with any missing items in the Big Five Inventory (BFI) or Depression Anxiety and Stress Scales-21 item (DASS-21) scales were imputed using the person-mean of available items (1.14% of BFI-N cases, 1.71% of BFI-E, 1.61% of BFI-O, 2.37% of BFI-A, 1.80% of BFI-C, and 5.03% of DASS-21).

A total of 1,055 participants were included in analyses. Five hundred sixty-two participants were recruited via the email link and 493 participated for research credit. The majority of participants were female, white, heterosexual, and never married. Less than half of the sample identified as an undergraduate student. The full demographic characteristics of the sample are listed in Table 1, including: sex assigned at birth, gender identity, race, sexual orientation, relationship status, highest level of education, English proficiency, and affiliation with the school (e.g. undergraduate, staff, faculty, etc.).

Individuals were permitted and encouraged to participate in multiple phases of data collection. During Phase 2 of data collection, 74 participants indicated that they had participated in Phase 1 of the study (7.01% of the total sample). During Phase 3 of data collection, 12 indicated that they participated in Phase 1, 13 participated in Phase 2, 47 participated in Phases 1 and 2, and 38 indicated that they participated in one other phase, but were unsure which one (Phase 3 repeaters *n* = 110; 10.43% of total sample). Accordingly, approximately one sixth of the total sample were repeat responders (*n* = 184, 17.44%).

### Measures

#### Personality Traits.

The FFM domains were assessed through the self-report Big Five Inventory (BFI; John & Srivastava, 1999). The scale is comprised of 44 items that begin with “I see myself as someone who…,” and the statements are rated on a 5-point Likert scale ranging from 1 (“disagree strongly”) to 5 (“agree strongly”). This scale produces summed scores for neuroticism (based on 8 items), extraversion (8 items), openness (10 items), agreeableness (9 items), and conscientiousness (9 items). The BFI has been validated across many cultures, languages, and the lifespan (e.g. Alansari, 2016; Benet-Martínez & John, 1998; Fossati et al., 2011). Within our sample, Cronbach’s alpha scores for neuroticism, extraversion, openness, agreeableness, and conscientiousness were *α* = .84, .84, .77, .77, and .83, respectively.

#### Distress.

The 21-item Depression Anxiety and Stress Scales-21 item (DASS-21) is a widely used tool that has evidenced validity in community settings and a variety of cultures (Antony et al., 1998; Lovibond & Lovibond, 1995; Tran et al., 2013). It is a self-report measure assessing symptoms of depression, anxiety, and stress over the past week. Summed scores can be interpreted to indicate normal to extremely severe levels of the assessed construct. Response options range on a four-point scale from 0 (“did not apply to me at all”) to 3 (“applied to me very much, or most of the time”). Examples of items include: “I felt that I had nothing to look forward to,” “I felt I was close to panic,” or “I found it difficult to relax.” In the present sample, Cronbach’s alpha was *α* = .94 for the total DASS score.

#### COVID-Specific Lifestyle Items.

Our research group generated several lifestyle items relevant to COVID-19 impact, based on collaborative discussion of factors that were likely to impact functioning. The response format for these items was broadly a dichotomous “yes” or “no.” The items included:

1. In the past week, have any children under your care had to stay home from school because of the Coronavirus (COVID-19)?
2. In the past week, have you been at home with school-aged children during your working hours?
3. Myself and any people that I live with have a comfortable amount of space inside the home (5-point Likert scale from strongly disagree to strongly agree).
4. Do you have any private outdoor space in which to spend time?
5. Has your employment security been significantly affected by the Coronavirus (COVID-19) outbreak?
6. Has your income been negatively affected by the Coronavirus (COVID-19) outbreak?

To mimic the dichotomous format of the majority of items, we dichotomized items 2 and 3. For item 2, “no” included responses “no” and “yes, as is routine,” while “yes” included the response “yes.” For item 3, strongly disagree and disagree were coded as “no,” agree and strongly agree coded as “yes,” and “neutral” was counted as a missing response (*n* = 74; 7.01%). This was done in order to be most conservative in the transformation, though all observed effects were retained when neutral responses were included in either group.

An additional question was aimed to assess potential behaviors such as media consumption in response to the pandemic, asking respondents to, “please indicate what percentage of time you spent doing the following during the past week: watching television to get information about COVID-19; talking to friends/relatives about COVID-19; consulting the internet for news about COVID-19; reading published accounts of people infected with COVID-19; trying to help those who were affected in some manner; going about your daily activities (work, school, errands); other.”

### Data Analysis

In accordance with the preregistration, we included the date of survey completion as an independent, continuous covariate in our analyses given uneven and overlapping participation across phases and recruitment samples. We did not operationalize time categorically by recruitment phase (i.e. 1, 2, or 3). Instead, “time” was entered as a “date variable” in SPSS, thus accounting for date and time of survey completion (e.g. 22-Mar-2020 14:49:55). This time variable served as a necessary control variable in the event that levels of distress systematically changed (i.e., decreased or increased) over the course of the pandemic and shelter-in-place orders. We chose to collapse across the two recruitment groups because any group differences would be confounded by differing time frames of data collection and varying numbers of undergraduates.

Bivariate zero-order correlations were examined for all study variables, including BFI scales, the DASS-21, and COVID-specific items. For our primary analyses, six hierarchical regression models with moderation were run to determine whether each COVID-specific item attenuated or amplified the effects of personality-level traits (i.e. BFI domain scales) on global distress (i.e. total DASS-21 scores) in individuals. To aid in interpretability of results, the BFI scores and the DASS-21 total score were standardized by converting to z scores. The continuous variable of time (i.e. the date and time of survey completion) was entered in Step 1 to determine if there was a significant effect of time on distress. The five domain scales with each moderator were entered in Step 2 for each of the moderated hierarchical regressions. Specifically, standardized global distress scores were regressed onto the COVID-specific item (unstandardized), BFI scales (standardized), and their interactions. To account for multiple comparisons for all BFI-by-COVID-item moderation hypotheses, we employed the False Discovery Rate (FDR) correction for *p*-value interpretation (Benjamini & Hochberg, 1995) within each model. Therefore, for each COVID-specific predictor, we corrected for six comparisons with FDR set at 5%, yielding a p-value cutoff beginning at .008. All main effects of BFI were evaluated using a *p*-value cutoff of *p* < .05. In accordance with our preregistration and in order to contextualize the probable robustness of our results, we performed a sensitivity power analysis to estimate the range of effects that we were powered to find in these moderation analyses.

Three additional hierarchical regressions without moderation were conducted as exploratory analyses. This was intended to simplify the analyses and preserve power after no moderators reached statistical significance. Due to the number of exploratory tests, we employed an FDR correction to each model to aid our p-value interpretation. For all analyses, time was entered in Step 1. For one exploratory hierarchical regression, DASS-21 scores were regressed onto the five personality domain scores at Step 2. We corrected for five comparisons within the model with FDR set at 5%, yielding a *p* cutoff beginning at .01. For another, DASS-21 scores were regressed on the six COVID items at Step 2. We corrected for six comparisons within the model with FDR set at 5%, yielding a *p* cutoff beginning at .008. For the final regression, DASS-21 scores were regressed on the domain scores *and* COVID item ratings at Step 2. We corrected for eleven comparisons within the model with FDR set at 5%, yielding a cutoff beginning at .005.

Because past research has indicated that personality predicts coping style (e.g. Ahern et al., 2002; Marshall et al., 2007), and that media consumption is a risk factor for distress following a large-scale trauma (e.g. Silver et al., 2002; Straud et al., 2015), we conducted a final exploratory analysis related to these topics. We examined how the domain scores predicted percentage of time in the past week spent engaging in COVID-related media consumption and activity (i.e. the final measure described above). To accomplish this, we regressed the percent time spent for each activity onto the BFI using linear regression. We corrected for five comparisons within each model with FDR set at 5%, yielding a cutoff beginning at .01.

## Results

Descriptive statistics and zero-order correlations of the BFI domain scores, total DASS score, and COVID-specific lifestyle items are presented in Table 2.

### Moderation Analyses (Table 3).

Six hierarchical regression analyses with moderation were conducted. Time was entered at Step 1. Step 2 included the domain scores, one of the six COVID lifestyle items, and five domain*COVID item interaction terms. After making the FDR correction, there were no statistically significant moderations in any of the analyses. Across all regressions, there were no statistically significant effects of time, which was entered at Step 1 (*ΔR*^2^ = 0.00 – 0.10%). The variables added at Step 2 explained a statistically significant amount of variance in all models across the six hierarchical regressions (*ΔR*^*2*^ = 28.8 – 30.5%; *p* = <.001 for all models). Across all of the COVID-specific lifestyle items and their interactions with personality traits, personality main effects alone accounted for almost all statistically significant effects and a vast majority of the explained variance at Step 2.

There were significant main effects of neuroticism for all six moderation analyses such that levels of neuroticism were positively associated with levels of distress, and neuroticism uniquely accounted for between 3.31% (inside space; *β* = 0.63, *p* = < .001) and 19.45% (homeschooling; *β* = 0.54, *p* = < .001) of the variance in distress (employment insecurity: *β* = 0.52, *p* = < .001; income insecurity: *β* = 0.52, *p* = < .001; child-work conflict: *β* = 0.55, *p* = < .001; outside space: *β* = 0.63, *p* = < .001). Significant main effects of openness were observed in the moderation analyses when items 1 and 2 (i.e. the childcare items) were included in the models such that higher levels of openness were associated with higher levels of distress (homeschooling: *β* = 0.68, *sr*^2^ = 0.32%, *p* = .029; child-work conflict: *β* = 0.082, *sr*^2^ = 0.41%, *p* = .014). Higher extraversion was associated with decreased distress for the item 4 moderation model, which assessed the availability of private outdoor space (*β* = −0.13, *sr*^2^ = 0.27%, *p* = .045). Higher agreeableness was also positively associated with distress for the homeschooling (item 1) moderation model (*β* = 0.64, *sr*^2^ = 0.27%; *p* = .049). Following the FDR correction, only two COVID items exhibited main effects: employment (item 5) and income (item 6) insecurity. Item 5 (employment insecurity) uniquely accounted for 1.19% of the variance in distress. (*β* = 0.11, *p* = <.001), and item 6 (income insecurity) uniquely accounted for 1.01% (*β* = 0.11, *p* = <.001). The endorsement of these items (i.e. indicating “yes”) was associated with a statistically significant increase in distress.

### Power analyses (Lane & Hennes, 2018, 2019).

As was preregistered, we conducted a series of power analyses to ascertain the power achieved and sensitivity to uncertainty for our obtained effect sizes of the moderation analyses. Specifically, we added and subtracted 10%, 20%, and 50% to each effect size to estimate the power curve of each effect size for samples between 100 and 1500 individuals in 50-person increments. Next, we inspected the magnitude of change in power given varying effect and sample size and flagged effects that evidenced insufficient power at observed or manipulated effect sizes (i.e., those with less than 80% power at our sample size). Given our sample’s parameters (n = 1055; 12 predictors, *α* = .05, *β* = .20), we were powered to find an average effect of Cohen’s f^2^ = .0075. Across models, neuroticism showed the most robust effects, with Cohen’s f^2^ values ranging from .04 (for COVID item 3, inside space) to .27 (for COVID item 1, homeschooling); even at 50% of neuroticism’s smallest observed effect size, estimated power was ≽ 90% given at least 500 observations. Next, the main effects of COVID items 5 (employment insecurity) and 6 (income insecurity) in their respective models evidenced adequately powered effects on global distress, where even at effect sizes reduced by 50%, we were powered at ≽ 80% to find their effects given our sample size. Lastly, the remaining effects of personality across models evidenced insufficient power to detect an effect at observed, and up to 50% increased, effect sizes at our sample size (power between 51% - 70%) with the exception of openness in the models including COVID items 1 (homeschooling) and 2 (child-work conflict), which reached 80% power at 50% increased effect size (f^2^ = .0075 - .0087). However, given that our models likely overestimated, rather than underestimated true population effects, neuroticism alone is likely the most reliable personality domain that influenced global distress in our study.

### Exploratory Analyses (Table 4).

Three additional hierarchical regressions without moderation were fit. The five domain scores were entered at Step 2 for the first exploratory analysis. Altogether, this model accounted for 28.7% of the variance in self-reported distress (*p* < .001). Compared to Steps 1 and 2 of all analyses in the present report, the largest ΔF value of them all was observed in Step 2 of this analysis (Δ*F*(5, 1044) = 83.99, *p* < .001). Similar to the moderation analyses, a main effect of neuroticism was observed such that higher neuroticism was associated with markedly more distress, and neuroticism uniquely accounted for a substantial 26.1% of the variance in distress (*β* = 0.55, *p* = <.001). There was also a main effect of agreeableness such that higher trait agreeableness was associated with higher distress, accounting for 0.49% of the variance in distress (*β* = 0.08, *p* = .010).

For another exploratory analysis, the six COVID lifestyle items were entered at Step 2. This model accounted for 4.5% of the total variance in distress (*p* <.001). Here we observed that having a comfortable amount of space at home and having private outdoor space were associated with lower distress scores, and these items uniquely accounted for 0.49% (*β* = −0.07, *p* = .030) and 1.00% (*β* =− 0.11, *p* = .001) of the variance in distress, respectively. Alternatively, experiencing a threat to employment or income security were associated with higher distress scores, and these uniquely accounted for 0.49% (*β* = 0.08, *p* = .022) and 0.81% (*β* = 0.10, *p* = .005) of the variance in distress, respectively. There were not statistically significant effects for the childcare items.

In an additional exploratory analysis, the five domain scores and six COVID lifestyle items were all entered at Step 2. Here, neuroticism, agreeableness, and employment insecurity each had unique positive associations with distress at a statistically significant level. Neuroticism uniquely accounted for 24.0% of the variance in distress (*β* = 0.54, *p* = <.001), agreeableness for 0.49% (*β* = 0.08, *p* = .007), and employment insecurity for 0.64% (*β* = 0.08, *p* = .012). Income insecurity accounted for 0.49%, but this did not reach statistical significance following FDR correction (*β* = 0.07, *p* = .028). Having private outdoor space was negatively associated with distress, and this accounted for 7.7% of the variance in distress (*β* = −0.08, *p* = .004). In this analysis that controlled for personality traits and other stressors, having a comfortable amount of space in the home and the childcare items were not associated with self-reported distress.

Finally, the linear regression analysis of time spent participating in COVID-related behaviors revealed most consistent associations with extraversion and conscientiousness. Extraversion was positively associated with consuming information via television (*β* = 0.13, *sr*^2^ = 1.4%, *p* < .001), discussing the pandemic with loved ones (*β* = 0.12, *sr*^2^ = 1.2%, *p* = < .001), and trying to help those affected by COVID (*β* = 0.09, *sr*^2^ = 0.72%, *p* = .006), while negatively associated with going about typical activities (*β* = −.14, *sr*^2^ = 1.6%, *p* < .001). Conscientiousness was positively associated with going about daily activities (*β* = 0.21, *sr*^2^ = 3.7%, *p* < .001), as well as negatively associated with talking to loves ones (*β* = −0.07, *sr*^2^= .45%; *p* = .029) and consulting the internet for COVID information (*β* = −0.08, *sr*^2^= .50%, *p* = .020). Neuroticism was only associated with a positive relationship of talking to loved ones (*β* = 0.10, *sr*^2^= .96%, *p* = .001), openness was negatively associated with consuming information via television (*β* = −0.09, *sr*^2^= .81%, *p* = .003), and agreeableness was positively associated with trying to help those affected (*β* = 0.09, *sr*^2^ = .67%, *p* = .007).

## Discussion

The COVID-19 pandemic was an acutely impactful event for individuals across the world. Beyond the direct illness and mortality consequences caused by the disease itself, the resulting stay-at-home period spurred a great deal of discomfort and distress (Bo et al., 2020; Galea et al., 2020; Pfefferbaum & North, 2020; Wallace et al., 2020). These circumstances created a natural experiment and an opportunity to understand how individual differences in personality, as well as in specific lifestyle factors, combine to influence distress and coping.

The present study provides a broad perspective of a significant public health stressor and its impacts on mental health across the early stages of governmental action and societal impact. Data collection commenced at the beginning of shelter-in-place orders and continued for two months, from days within the first COVID-related death in the geographic locale of data collection to days within reaching 100,000 COVID-19 deaths in the USA. In addition to the broad temporal scope of data collection, we sampled a large, diverse sample from within a heterogeneous community. Accordingly, these findings offer increased generalizability, particularly in comparison to the purely undergraduate or clinical samples common in research of personality and psychiatric distress.

Open science principles including preregistration were used in the current study. The findings presented here that did not reach statistical significance are informative nonetheless, particularly because findings contrary to well-researched hypotheses warrant thoughtful consideration. Indeed, several effects that we hypothesized were not supported by the data.

As predicted, certain personality traits had a main effect on distress. Zero-order correlations revealed that all domains except openness had statistically significant correlations with distress, as was expected (i.e. all correlations were negative except for that of neuroticism). However, when entered simultaneously, it was clear that neuroticism was the predominant factor that held across all moderation and exploratory analyses.

The particularly strong effect of neuroticism on distress was highly consistent with past research (e.g. Brow & Silver, 2009; Kotov et al., 2010; Naragon-Gainey & Simms, 2017). This finding is probably best understood as probable construct overlap between the DASS-21 and the neuroticism items of the BFI, as well as genuine exacerbation of distress for those higher in neuroticism. As research has shown, those higher in neuroticism tend to both experience more stressful events and report more distress from those events (e.g. Griffith et al., 2010; Kopala-Sibley et al., 2016; Kotov et al., 2017) These effects suggest that the psychological harm of the COVID-19 pandemic is more severe for those higher in neuroticism, and increased support for these individuals is necessary. We cannot know for sure if the observed link between neuroticism and distress in the context of the pandemic goes above and beyond their baseline association. In the present sample, the regression coefficients for the association between neuroticism and distress ranged from *β* = 0.54 to *β* = 0.63 in the preregistered analyses and from *β* = .54 to *β* = .55 in the exploratory analyses. In a meta-analysis of ten prospective community cohort studies totaling 117,899 participants, cross-sectional associations between neuroticism and depressive symptoms yielded a pooled regression coefficient of *β* = 0.39 (Hakulinen et al., 2015). This could suggest that the pandemic has an amplifying effect on the link between neuroticism and distress, but this is an imperfect and approximate comparison, and future research incorporating baseline measurements is needed.

Our hypothesis that extraversion would be negatively associated with distress was broadly not supported, with no observed effects for five of the six moderations nor for any of the exploratory analyses. We based this hypothesis on past research that has identified extraversion as a clear protective factor from psychological stress (e.g. Clark et al., 1994; McCrae & Costa, 2013; Straud et al., 2015). But the circumstances of this study are unique from past research, particularly because the participants were (ostensibly) engaging in social isolation. It is possible that the pandemic might be more difficult for those who are more sociable, and the protective components of extraversion are neutralized by the deficits of social isolation. The exploratory analysis of time spent on COVID-related activities could offer some insight into this unexpected finding as well; extraversion was positively associated with increased consumption of information via television, discussing the pandemic with loved ones, and helping those affected by COVID-19, while negatively associated with going about typical activities of daily life. It seems that higher extraversion was associated with increased participation both behaviorally and mentally with the pandemic, and this increased exposure and interference with daily life could certainly lead to increased distress.

Also in contrast to our hypotheses, agreeableness was positively associated with distress even when adjusting for the other domains. This finding was consistent across exploratory analyses, though a main effect of agreeableness was observed only in one moderation analysis. This could be explained by the relatively small amount of variance accounted for by agreeableness, which consistently accounted for less than one percent of variance in distress. It is hard to know exactly what could explain this residual variance, but it might have to do with the demonstrated, positive relationship agreeableness shares with empathy and emotional responsiveness—over and above other FFM traits (Graziano et al., 2007; Tobin et al., 2000). It is conceivable that those higher in agreeableness were suffering from empathy fatigue given the negative state of the world, and particularly when suffering, death, and disease was so regularly portrayed in the media. This empathy fatigue might have been heightened further as anti-lockdown protests erupted across the country. These possibilities are somewhat corroborated through the finding that agreeableness was positively associated with helping those affected by COVID-19. To further elucidate the meaning of these findings, it could help to survey the association between distress and the facets of agreeableness (i.e. trust, straightforwardness, altruism, compliance, modesty, tender-mindedness), but facet-level scores were not possible given our measure.

There were no observed effects pertaining to conscientiousness and distress across moderation and exploratory analyses. This could be explained by the finding that conscientiousness was positively associated with continuing to go about typical activities of daily life, thus suggesting that more conscientiousness was associated with less COVID-related change to lifestyle. Higher conscientiousness could be associated with the discipline and dedication necessary to maintain professional and personal goals in spite of an impact so disruptive as the COVID-19 pandemic. Again, a facet-level analysis would have assisted in the interpretation of these findings. Finally, openness exhibited a main effect in the moderation analyses of both childcare items such that higher openness was associated with higher distress when accounting for childcare burden. However, there were no observed effects of openness for the consolidated exploratory analyses. It is therefore unclear what role openness plays in the relationship between distress, lifestyle change, and personality.

Certain lifestyle changes also influenced level of distress, though this was not the case for all lifestyle factors we surveyed. Threat to employment or income security and insufficient indoor or outdoor space were all significantly and positively associated with distress at the zero-order level. Unsurprisingly, the experience of negative employment impact was most associated with increased distress across all analyses. In addition, having adequate space, particularly *outdoor* space, had a unique buffering effect such that those who endorsed having enough space were less distressed. This effect was particularly pronounced in the exploratory analyses. It stands to reason that those without access to private outdoor space were differentially impacted by shelter-in place because remaining indoors was an added burden. This finding is particularly relevant for informing policy in the future. Much attention was paid to loss of employment or income at the legislative level, but the importance of outdoor space was widely neglected.

Surprisingly, the child- and family-care stressors did not have a statistically significant effect—not even at the zero-order level. This could reflect a low base rate of those with children in our sample (particularly among undergraduate and graduate participants), or perhaps even that guardians were too preoccupied to take part in the survey at all. Unfortunately, specific information on parental status was not collected, such as the ages or number of children in the respondents’ care. We take these findings to be limited and preliminary, and more work in this area is needed.

The data did not support our hypotheses regarding potential moderation effects of the lifestyle factors on the relation between personality and distress. This suggests that the effects of personality and lifestyle may be additive and not compounding. It is worth noting that interactions of the magnitudes we observed would be considered underpowered given the obtained sample; even with a sufficient sample size, they would amount to very small effects (i.e. <0.05% of variance). Another concern is the precision of our measurements. Specifically, the lifestyle factors were determined from one-item indicators, and their individual reliability is inherently limited. Nonetheless, there is insufficient prior research concerning the potential moderating factors on personality in the face of an acute, wide-spread stressor. One study reported the statistically significant interaction between exposure level and personality on trauma symptoms (Kopala-Sibley et al., 2016), and other research into disasters that included interactions were even less relevant to the constructs of the present discussion (e.g. Ahern et al., 2002; Marshall et al., 2007; Silver et al., 2002). Taken together, while the interactions between personality and COVID-related lifestyle items were not supported, this does not suggest this is a closed topic given the limitations of the study and the paucity of research into the subject.

The effects of linearly-measured time did not reach statistical significance, suggesting that time did not control or account for distress over the two-month period of data collection. If there was an effect of time, this would have suggested that distress broadly increased or decreased over the course of data collection. But the reality seems to be that there is no clear trajectory of distress, and the individual experience is likely what varies. This emphasizes the importance of consistent support and resources throughout the pandemic; the situation does not necessarily get better or worse as time goes on.

### Limitations.

Due to the time-sensitive nature of assembling the study, a few potentially informative items were overlooked for inclusion in the assessment battery. Specific to the data reported here, we did not assess age of the participants. This was done with time constraints in mind as we balanced the desire for timely study launch against the delay to receive IRB approval for potentially identifiable data. We also did not collect specific information about parental status (e.g. number of children, ages of children), and this could have allowed for a more nuanced picture of the findings related to the childcare items. Further, individuals could participate in multiple phases of data collection. Due to the non-identifying nature of data collection, we did not have a way of connecting the data to make longitudinal, within-person statistical comparisons. About one sixth of the total sample were repeat responders, making this more aligned with a cross-sectional rather than longitudinal study. Additionally, the present study did not include pre-pandemic measures that would have allowed the assessment of more direct causal impacts of personality on distress. Further, in an effort to maximize survey completion, the investigators prioritized brevity in measure selection. Opting for a FFM measure longer than the BFI could have permitted the determination of facet scores. Opting for the DASS-21 instead of the full DASS limited the amount of clinical information provided by the outcome variable, but prior research has indicated that the DASS-21 might actually be preferable from a psychometric standpoint (Antony et al., 1998). Finally, active data collection lasted over a period of 65 days, and circumstances changed rapidly during the pandemic—certainly within a week and sometimes within days or hours. Ideally, data collection could have taken place over a more restricted time interval, but the prioritization of generating a sufficient sample size limited this effort.

### Suggestions for Future Preregistrations.

There is always substantial risk of oversight and omission when developing a study in a short time-frame, and there are several ways the authors could have improved this study’s preregistration. We identify these shortcomings in the hopes that scientists collecting data during timely events in the future might learn from our oversights. In hindsight, the preregistration suffered from lack of detail. A straightforward recommendation is to include the method of accounting for multiple comparisons. The *p* value corrections for exploratory analyses or multiple comparisons were not preregistered for the present report. Additionally, no matter a scientist’s confidence in hypothesized effects, the authors suggest incorporating the possibility of unexpected results into analytical decision trees and considering the analyses that might result from such outcomes. The exploratory analyses in the present report might have been planned a priori if the authors had done so. Further, the authors failed to include the directionality of hypothesized effects as well as the participant exclusion criteria in the preregistration. As a final recommendation, researchers should ensure that the scale items generated by their research team are measured on the same scale format. This would have allowed us to avoid dichotomizing the Likert assessment of sufficient indoor space. In sum, preregistering a study during a moment of empirical urgency is commendable, but rigorous detail in these plans is the ultimate achievement.

### Constraints of Generalizability (Simons et al., 2017).

We would expect our findings to generalize to other measures of psychological distress and Big Five personality traits given the extensive validation of the DASS-21 and BFI. Our assessment of COVID-related lifestyle effects involved single-item, dichotomous constructs that our research team generated, so we cannot be sure the findings related to lifestyle effects would generalize to other measures of such constructs. Further, the findings are applicable to college- and working- aged adults, and although the affiliated university has a substantial proportion of international and transcontinental affiliates, we cannot be sure that these findings would generalize outside the Midwestern United States.

### Strengths.

Strengths of this study include a more inclusive sample than a typical study from a university-setting; instead of only recruiting undergraduate students seeking academic research credit for introductory classes, we broadly recruited undergraduates, graduate students, faculty, and staff from a variety of colleges across campus. The collection of data across time is also a strength: individuals were sampled at the onset of national lockdown, approximately 3 weeks after lockdown, and as reopening was initiated locally. Further, data collection began relatively early in the pandemic’s progression in the United States and included several significant events such as the implementation of shelter-in-place orders across the country. Because this study offers a look into an earlier stage of this the pandemic, it is our hope that it will be particularly beneficial in understanding how early responses to this devastating pandemic inform the long-term impacts we are beginning to observe.

## Conclusion

A key takeaway of the present study is that personality and lifestyle factors both play independent, notable roles in predicting psychological distress during a pandemic. In particular, these findings help to identify individuals who might be at greater risk for distress. While neuroticism is an expected (if not obvious) risk factor, agreeableness is another potential unique risk factor, though the latter effect was almost exclusively observed in the exploratory analyses. The consideration of lifestyle factors is also essential. These findings especially highlight the link between employment and income uncertainty with anxiety, depression, and stress. This emphasizes the need for mental health outreach programs for less secure or furloughed employees, as well as the need for ongoing legislative action to offer support for these individuals. Insufficient indoor and outdoor space are other potential risk factors in need of increased attention.

As COVID-19 has disrupted multiple aspects of individuals’ lives across the world, the resulting stress could likely continue to intensify. Our hope is that this information can be used to inform response and action at a variety of levels, from within the home to governmental bodies, and from the current pandemic to future public health events. Beyond the pandemic, this research further emphasizes the utility of personality trait models, and in particular how these constructs relate to psychological problems—including even transient or uncharacteristic distress.

## Supplementary Material

1

Peer Review and Communication History. Docx Response to Reviewers. PDF

## Figures and Tables

**Table 1.** Demographic characteristics of total sample (n = 1,055)*.

	*n*	%
Sex assigned at birth		
Female	658	62.7
Male	390	37.2
Intersex	1	0.1
Gender identity		
Female	658	62.7
Male	390	37.2
Non-Binary	1	0.1
Race		
White	791	75.9
Asian	151	14.5
Latinx	44	4.2
Other	29	2.8
Black	27	2.6
Sexual orientation		
Straight/heterosexual	945	91.3
Bisexual	58	5.6
Gay or lesbian	24	2.3
Other	8	0.8
Relationship status		
Never married	575	57.0
Married	318	31.5
Living with partner	75	7.4
Divorced	27	2.7
Widowed	7	0.7
Separated	7	0.7
Education		
Some college	398	37.9
Completed graduate school	310	29.5
Some graduate school	144	13.7
Graduated HS/received GED	104	9.9
Graduated 4-year college	90	8.6
Graduated 2-year-college	4	0.4
University affiliation^a^		
Undergraduate student^b^	524	—
Faculty	181	—
Staff	185	—
Graduate student	201	—
Alumni	54	—
Administration	22	—
Parent	14	—

* Answering demographic questions was not required. There are missing data in each category (sex, *n* = 6; gender, *n* = 6; race, *n* = 13; orientation, *n* = 20; relationship, *n* = 46; education, *n* = 5).

a Estimate. Participants instructed to select all that applied, does not total 100% (none selected: *n* = 14).

b Includes both former and current undergraduates.

**Table 2.** Descriptive statistics and zero-order correlations of the BFI domains, DASS total score, and COVID-specific lifestyle items (C-items).

Scale	*M*	*SD*	1	2	3	4	5	6	7	8	9	10	11	12
1. DASS Total	27.77	24.4	—											
2. BFI-N	23.67	6.43	.53**	—										
3. BFI-E	25.55	6.22	−.13**	−.23**	—									
4. BFI-O	35.21	5.96	−.02	−.12**	.23**	—								
5. BFI-A	34.04	5.41	−.09**	−.29**	.22**	.18**	—							
6. BFI-C	33.51	5.94	−.14**	−.27**	.18**	.08**	.35**	—						
7. Homeschool	0.22	0.41	−.01	−.09**	.11**	.02	.07*	.09**	—					
8. Child-Work Conflict	0.32	0.47	−.03	−.08*	.05	−.01	.05	.05	.60**	—				
9. Inside Space	0.92	0.27	−.12**	−.08*	.08**	−.04	−.02	.07*	.03	.03	—			
10.Outdoor Space	0.80	0.40	−.11**	−.08*	.12**	.01	.05	.10**	.11**	.16**	.30**	—		
11.Job insecurity	0.27	0.44	−.14**	.05	.08**	−.00	−.02	−.13**	.03	.02	−.09**	−.01	—	
12.Income insecurity	0.30	0.46	.15**	.09**	.11**	.02	−.05	−.12**	.04	.10**	−.12**	.01	.49**	—

*Note*. DASS total = 21-item Depression, Anxiety, Stress Scales. BFI = Big Five Inventory; N = Neuroticism, E = Extraversion, O = Openness, A = Agreeableness, C = Conscientiousness. C-Item = COVID-Specific Lifestyle Item. *n* = 1055. All DASS-21 scores are doubled for interpretation through DASS-42 guidelines. All C-items are based on a dichotomous response format where 0 = no and 1 = yes.

* (*p* = <.05)

** (*p* = <.01)

**Table 3.** Hierarchical moderation regressions predicting distress scores on the DASS-21.

	Homeschool				Child-Work Conflict			
Step 1	*B*	*95*% *CI*	*SE*	*sr*	*B*	*95*% *CI*	*SE*	*sr*
Time	0.00	−183.76, 680.41	0.00	−0.03	0.00	−194.56, 667.72	0.00	−0.03
	*R*^*2*^ = 0.00, *ΔF* = 1.27				*R*^*2*^ *=* 0.00, *ΔF =* 1.16			
Step 2:	*B*	*95*% *CI*	*SE*	*sr*	*B*	*95*% *CI*	*SE*	*sr*
Neuroticism	**0.51**	**0.45, 0.57**	**0.03**	**0.44** ***	**0.52**	**0.46, 0.59**	**0.03**	**0.41** ***
Extraversion	−0.03	−0.09, 0.03	0.03	−0.02	−0.04	−0.11, 0.02	0.03	−0.03
Openness	**0.07**	**0.01, 0.13**	**0.03**	**0.06** *	**0.08**	**0.02, 0.14**	**0.03**	**0.06** *
Agreeableness	**0.06**	**< 0.00, 0.13**	**0.03**	**0.05** *	0.04	−0.03, 0.11	0.04	0.03
Conscientiousness	−0.04	−0.10, 0.02	0.03	−0.03	−0.05	−0.12, 0.02	0.03	−0.04
COVID Item	0.06	−0.06, 0.19	0.06	0.03	0.02	−0.09, 0.13	0.05	0.01
COVID Item*N	0.03	−0.10, 0.16	0.07	0.01	−0.01	−0.12, 0.10	0.06	−0.01
COVID Item*E	0.01	−0.12, 0.14	0.07	0.00	0.06	−0.05, 0.17	0.06	0.03
COVID Item*O	−0.08	−0.20, 0.04	0.06	−0.03	−0.12	−0.23, −0.01	0.06	−0.06
COVID Item*A	0.04	−0.10, 0.18	0.07	0.02	0.09	−0.03, 0.21	0.06	0.04
COVID Item*C	0.09	−0.05, 0.23	0.07	0.03	0.10	−0.01, 0.22	0.06	0.05
	***R***^***2***^ ***=* 0.29, *ΔR***^***2***^ *=* 0.29, *ΔF =* 37.95***				***R***^***2***^ ***=* 0.30, *ΔR***^***2***^ *=* 0.30, *ΔF =* 39.50***			
	Inside Space				Outside Space			
Step 1	*B*	*95*% *CI*	*SE*	*sr*	*B*	*95*% *CI*	*SE*	*sr*
Time	0.00	−262.04, 626.93	0.00	−0.03	0.00	−238.34, 618.84	0.00	−0.03
	*R*^*2*^ *=* 0.00, *ΔF =* 0.65				*R*^*2*^ *=* 0.00, *ΔF =* 0.76			
Step 2:	*B*	*95*% *CI*	*SE*	*sr*	*B*	*95*% *CI*	*SE*	*sr*
Neuroticism	**0.59**	**0.42, 0.76**	**0.09**	**0.18** ***	**0.60**	**0.49, 0.70**	**0.05**	**0.28** ***
Extraversion	0.02	−0.18, 0.21	0.10	0.00	**−0.12**	**−0.25, < −0.00**	**0.06**	**−0.05** *
Openness	0.04	−0.16, 0.24	0.10	0.01	0.07	−0.06, 0.19	0.06	0.03
Agreeableness	0.07	−0.14, 0.29	0.11	0.02	0.03	−0.10, 0.16	0.07	0.01
Conscientiousness	0.13	−0.08, 0.35	0.11	0.03	0.01	−0.10, 0.13	0.06	0.01
COVID Item	*−0.27*	*−0.48, −0.06*	*0.11*	*−0.07* ^a^	*−0.14*	*−0.27, −0.01*	*0.07*	*−0.06* ^a^
COVID Item*N	−0.09	−0.27, 0.09	0.09	−0.03	−0.10	−0.27, 0.02	0.06	−0.04
COVID Item*E	−0.05	−0.25, 0.15	0.10	−0.01	*0.13*	*−0.01, 0.26*	*0.07*	*0.05* ^a^
COVID Item*O	0.01	−0.20, 0.21	0.10	0.00	−0.04	−0.17, 0.10	0.07	−0.01
COVID Item*A	−0.01	−0.23, 0.21	0.11	0.00	0.06	−0.09, 0.20	0.07	0.02
COVID Item*C	−0.15	−0.37, 0.07	0.11	−0.04	−0.03	−0.16, 0.10	0.07	−0.01
	***R***^***2***^ ***=* 0.30, *ΔR***^***2***^ *=* 0.30, *ΔF =* 36.91***				***R***^***2***^ ***=* 0.30, *ΔR***^***2***^ *=* 0.30, *ΔF =* 40.47***			
	Job Insecurity				Income Insecurity			
Step 1	*B*	*95*% *CI*	*SE*	*sr*	*B*	*95*% *CI*	*SE*	*sr*
Time	0.00	−214.82, 647.03	0.00	−0.03	0.00	−212.83, 648.38	0.00	−0.03
	*R*^*2*^ *=* 0.00, *ΔF =* 0.97				*R*^*2*^ *=* 0.00, *ΔF =* 0.97			
Step 2:	*B*	*95% CI*	*SE*	*sr*	*B*	*95% CI*	*SE*	*sr*
Neuroticism	**0.49**	**0.43, 0.55**	**0.03**	**0.41** ***	**0.49**	**0.42, 0.55**	**0.03**	**0.40** ***
Extraversion	−0.03	−0.09, 0.03	0.03	−0.03	−0.06	−0.12, 0.01	0.03	−0.05
Openness	0.02	−0.04, 0.08	0.03	0.01	0.03	−0.03, 0.10	0.03	0.03
Agreeableness	0.05	−0.02, 0.11	0.03	0.04	0.04	−0.02, 0.11	0.03	0.03
Conscientiousness	0.01	−0.06, 0.07	0.03	0.01	0.03	−0.04, 0.09	0.03	0.02
COVID Item	**0.24**	**0.13, 0.36**	**0.06**	**0.11** ***	**0.22**	0.11, 0.33	**0.06**	**0.10** ***
COVID Item*N	0.07	−0.05, 0.19	0.06	0.03	0.07	**−0.04, 0.19**	0.06	0.03
COVID Item*E	−0.03	−0.15, 0.08	0.06	−0.01	0.06	−0.05, 0.17	0.06	0.03
COVID Item*O	0.08	−0.04, 0.19	0.06	0.03	0.04	−0.07, 0.15	0.06	0.02
COVID Item*A	0.09	−0.03, 0.22	0.07	0.04	0.09	−0.03, 0.21	0.06	0.04
COVID Item*C	−0.02	−0.14, 0.10	0.06	−0.01	−0.09	−0.21, 0.02	0.06	−0.04
	***R***^***2***^ ***=* 0.31, *ΔR***^***2***^ *=* 0.31, *ΔF =* 41.21***				***R***^***2***^ ***=* 0.31, *ΔR***^***2***^ *=* 0.30, *ΔF =* 41.16***			

*Note. B* = unstandardized estimate. *SE* = standard error. *sr* = semi-partial correlation. Δ*R^2^* = change in *R^2^* from Step 1 to Step 2.

* *p* < .05,

** *p* < .01,

*** *p* < .001

a non-significant following FDR correction

**Table 4.** Exploratory regression analyses predicting distress scores on the DASS-21.

	Domains Only				COVID Items Only				Domains & COVID Items			
Step 1	*B*	*95*% *CI*	*SE*	*sr*	*B*	*95*% *CI*	*SE*	*sr*	*B*	*95*% *CI*	*SE*	*sr*
Time	0.00	−213.50, 646.61	0	−0.03	0.00	−278.09, 617.09	0.00	−0.02	0.00	−250.16, 643.13	0.00	−0.03
	*R*^*2*^ = 0.00, *ΔF* = 0.98				*R*^*2*^ = 0.00, *ΔF* = 0.55				*R*^*2*^ = 0.00, *ΔF* = 0.75			
Step 2:	*B*	*95% CI*	*SE*	*sr*	*B*	*95% CI*	*SE*	*sr*	*B*	*95% CI*	*SE*	*sr*
Neuroticism	**0.52**	**0.46, 0.57**	**0.03**	**0.51** ***	-	-	-	-	**0.50**	**0.44, 0.55**	**0.03**	**0.49** ***
Extraversion	−0.02	−0.08, 0.03	0.03	−0.02	-	-	-	-	−0.05	−0.11,< 0.00	0.03	−0.05
Openness	0.04	−0.01, 0.09	0.03	0.04	-	-	-	-	0.05	−0.01, 0.10	0.03	0.05
Agreeableness	**0.07**	**0.02, 0.13**	**0.03**	**0.07** **	-	-	-	-	**0.08**	**0.02, 0.14**	**0.03**	**0.07** **
Conscientiousness	−0.01	−0.07, 0.04	0.03	−0.01	-	-	-	-	0.01	−0.04, 0.07	0.03	0.01
Homeschool	-	-	-	-	0.03	−0.15, 0.21	0.09	0.01	0.07	−0.08, 0.23	0.08	0.03
Child-Work Conflict	-	-	-	-	−0.03	−0.19, 0.13	0.08	−0.01	0.03	−0.11, 0.16	0.07	0.01
Inside Space	-	-	-	-	**−0.25**	**−0.48, −0.03**	**0.12**	**−0.07** *	−0.13	−0.32, 0.07	0.10	−0.04
Outside Space	-	-	-	-	**−0.27**	**−0.44, −0.11**	**0.08**	**−0.10** **	**−0.20**	**−0.34, −0.06**	**0.07**	**−0.08** **
Job Insecurity	-	-	-	-	**0.18**	**0.03, 0.33**	**0.08**	**0.07** *	**0.17**	**0.04, 0.30**	**0.07**	**0.07** *
Income Insecurity	-	-	-	-	**0.22**	**−0.07, 0.37**	**0.08**	**0.09** **	*0.14*	*0.02, 0.27*	*0.07*	*0.06* ^a^
	***R***^***2***^ ***=* 0.29, *ΔR***^***2***^ *=* 0.29, *ΔF =* 83.99***				***R***^***2***^ ***=* 0.05, *ΔR***^***2***^ *=* 0.05, *ΔF =* 8.46***				***R***^***2***^ ***=* 0.32, *ΔR***^***2***^ *=* 0.32, *ΔF =* 40.08***			

*Note. B* = unstandardized estimate. *SE* = standard error. *sr* = semi-partial correlation. Δ*R^2^* = change in *R^2^* from Step 1 to Step 2.

* *p* < .05,

** *p* < .01,

*** *p* < .001

a non-significant following FDR correction

## Data Availability

All the stimuli, presentation materials, participant data, and analysis scripts can be found on this paper’s project page on OSF: https://doi.org/10.17605/OSF.IO/5HR2T.
