# Supplementary material for: The Impact of Personality and Lifestyle Change on Distress During the COVID-19 Pandemic
Source: Collabra Psychol. Author manuscript; Available in PMC 2026 Jan 2. (PMC12757131; doi:10.1525/collabra.19525)
Supplement: 1 [file NIHMS2131671-supplement-1.pdf]

SUPPLEMENTARY MATERIALS

Response to reviewers

Download: https://collabra.scholasticahq.com/article/19525-the-impact-of-personality-and-lifestyle-change-on-distress-during-the-covid-19-pandemic/attachment/51766.pdf?auth_token=lCS7jFBh3EaA3DjS-cxo

Peer review history

Download: https://collabra.scholasticahq.com/article/19525-the-impact-of-personality-and-lifestyle-change-on-distress-during-the-covid-19-pandemic/attachment/51767.docx?auth_token=lCS7jFBh3EaA3DjS-cxo

Downloaded from http://online.ucpress.edu/collabra/article-pdf/7/1/19525/456085/collabra_2021_7_1_19525.pdf by guest on 29 April 2021
